# Characterization of Low-Molecular-Weight Collagen from Korean Native Chicken Feet Hydrolyzed Using Alcalase

**DOI:** 10.4014/jmb.2212.12047

**Published:** 2023-01-19

**Authors:** Heedong Woo, Gyeong A Jeong, Hyunwook Choi, Chang Joo Lee

**Affiliations:** 1 SCM Division, ASK COMPANY, Suseong, Daegu 42176, Republic of Korea; 2 Department of Food Science and Biotechnology, Wonkwang University, Iksan 54538, Republic of Korea; 3 Department of Functional Food and Biotechnology, Jeonju University, Jeonju 55069, Republic of Korea

**Keywords:** Alcalase, Korean native chicken foot, low-molecular-weight collagen

## Abstract

The aims of this study were to optimize the preparation of low-molecular-weight collagen using a proteolytic enzyme (alcalase) derived from the feet of Korean native chickens, and to characterize the process of collagen hydrolysis. Foreign bodies from chicken feet were removed using ultrasonication at 28 kHz with 1.36 kW for more than 25 min. The hydrolytic pattern and molecular weight distribution of enzyme-treated collagen from chicken feet were analyzed using sodium dodecyl sulfate-polyacrylamide gel electrophoresis and high-performance liquid chromatography, respectively. Ideally, chicken feet should be treated at 100°C for 8 h to obtain a high collagen content using hot water extraction. The collagen content of the chicken foot extract was 13.9 g/100 g, and the proportion of low-molecular-weight collagen increased with increasing proteolytic enzyme concentration and reaction time. When treated with 1% alcalase, the average molecular weight of collagen decreased rapidly to 4,929 Da within 5 h and thereafter decreased at a slower rate, reaching 4,916 Da after 7 h. Size exclusion chromatography revealed that low-molecular-weight collagen peptides of approximately 1,000–5,000 Da were obtained after hydrolysis with 1% alcalase for 1 h.

## Introduction

Social development associated with economic growth has had a pronounced influence on global economic wealth and resulted in an increase in interest in quality of life and life expectancy [1]. In Korea, this has been reflected by an upward trend in the functional health food market, in which the Ministry of Food and Drug Safety estimated it to have increased by 16.3% in 2020 compared with the previous year and recorded an average growth rate of 14.4% over the past three years [2]. This growth in demand for functional foods has provided an impetus for studies on the functionality of diverse food constituents and their industrial applications.

Collagen is a fibrous, insoluble protein found primarily in the skin, cartilage, and connective tissues. Recently, the demand for collagen peptides has been growing owing to their beneficial effects on skin health [3]. Although natural polymers are the main components of skin and hair, the main component of the skin is collagen, which is found in fish, pigs, and birds. In human skin, collagen is synthesized by fibroblasts as procollagen and subsequently converted to mature collagen molecules [4]. Collagen has been widely studied for its ability to enhance skin elasticity and retard its aging process [5]. Based on peptide secondary structures, collagen can be classified into approximately 28 types. Type I collagen is the predominant form in humans and accounts for over 90% in different tissues, such as skin, bones, and tendons [6]. Type II collagen is a unique component that supports healthy joints. Daily supplementation with 40 mg type II collagen enhances joint function and flexibility in healthy subjects, as demonstrated by greater knee extension. Additionally, it can alleviate joint pain that occasionally arises from strenuous exercise, prolonging periods of pain-free exertion [7]. Naturally derived collagen peptides are typical constituents found in numerous cosmetic products and are used as nutritional supplements with skin-related health benefits. Collagen peptides are characterized by a wide range of lengths and a high proportion of the amino acids, hydroxyproline, glycine, and proline, which are produced by the enzymatic hydrolysis of native collagen extracted from animal connective tissues [8]. Generally, from the perspective of molecular analysis, collagen contains 30% glycine, 25% proline and hydroxyproline, and 45% other amino acids. Glycine, proline, and hydroxyproline account for approximately 57% of total amino acids in the collagen structure [9], of which hydroxyproline comprises 12–14% (w/w) and is widely used for collagen quantification [10]. Clinically, oral supplementation with collagen peptide increased skin hydration after eight weeks of intake and the density of collagen in the dermis after four weeks [8]. A further clinical study showed that low-molecular-weight peptides are associated with high levels of amino acid digestion and absorption in the intestines [11]. Consumers prefer low-molecular-weight collagens among the wide range of available collagens [3]. In particular, commercial collagen peptides derived from fish scales have a molecular weight distribution ranging from 2,000 to 20,000 Da, which tends to be lower than that of animal collagen [12].

Natural sources of collagen include bovine, porcine, marine, ovine, and avian species [13]. Among the latter, chicken feet can be used as a potential source of collagen. Although not widely consumed, they contain important nutrients and have essential health benefits [14]. Although the application of chicken foot collagen in the food and pharmaceutical industries has been facilitated by acidic, alkaline, and enzymatic hydrolysis methods [15, 16], extraction using acid- or alkaline-based methods generates large volumes of acidic/alkaline wastewater, thereby resulting in a range of environmental problems, including pollution and high process costs associated with wastewater treatment [17]. Consequently, to reduce environmental pollution and increase bioavailability, there has been a shift toward the production of low-molecular-weight chicken foot collagen using enzymatic processes. In this study, we investigated the characteristics of low-molecular-weight collagen derived from Korean chicken feet and hydrolyzed using a serine endoprotease.

## Materials and Methods

### Materials

Chicken foot samples were prepared using the feet of Korean native chickens (*Gallus gallus domesticus*) purchased from Harim Co., Ltd. (Republic of Korea), which had been slaughtered on the same day. The enzyme used for the hydrolysis of chicken foot collagen was serine endopeptidase alcalase (E.C. 3.4.21.62, 2.5 AU-A; Novozymes Co., Denmark). All other chemicals used were of analytical grade.

### Methods

**Elimination of Foreign Bodies in Chicken Feet Using Ultrasonication**

Blood and foreign bodies in chicken feet were removed using an ultrasonic cleaner (AY-1500; Anyone, Korea). The ultrasonic frequency was 28 kHz, power 1.36 kW, and sonication periods 10, 15, 20, 25, and 30 min. The turbidity of washing water was measured at 600 nm using a spectrophotometer (UV-1800; Shimadzu, Japan). Test samples were hermetically sealed and stored at -18°C. The samples were analyzed in triplicate.

### Chicken Foot Collagen Extraction

Collagen was extracted from chicken feet by heating the sample for 3, 5, 7, and 9 h at 100°C with double-purified water. The water evaporated during the heating of the extraction solution was added to purified water, and the amount of crude protein in the extraction solution was measured with respect to heating time. The extract solution obtained was concentrated to 50% of the initial volume and passed through a 150-mesh sieve. The samples were analyzed in triplicate.

### Measurement of Crude Protein and Collagen Contents

The crude protein content of extracted chicken foot collagen was analyzed using the micro-Kjeldahl method [18] and calculated using a nitrogen transport coefficient of 6.25. Hydroxyproline quantification was performed using the methods described by Kolar [19] and Edwards and O`Brien [21]. To calculate the collagen content, we applied a coefficient of 8.0. We added 6 N HCl to 1 g of the samples, followed by hydrolysis for 16 h in a dry oven at 105°C. The hydrolysates were diluted and mixed with 1 ml Chloramine T solution, to which 1 ml color reagent was added. To evaluate the hydroxyproline content, the absorbance of the chilled sample solution was recorded at 550 nm using a UV-1080 spectrometer (Shimadzu Co.). The samples were analyzed in triplicate.

### Enzymic Preparation of Low-Molecular-Weight Collagen

Chicken foot collagen was pretreated with 0.1 N HCl at pH 6.5–7.0, followed by the addition of alcalase at 0.1%and 1.0% (w/w) relative to the chicken foot collagen extract. The enzyme-based hydrolysis reaction was carried out for 0, 1, 2, 3, 5, and 7 h at 60°C and stopped by boiling at 100°C for 15 min.

### Hydrolysis Pattern and Molecular Weight Distribution of Enzyme-Treated Collagen

The pattern of protein hydrolysis and molecular weight of enzyme-treated chicken foot collagen were analyzed using sodium dodecyl sulfate-polyacrylamide gel electrophoresis (SDS-PAGE) and high-performance liquid chromatography (HPLC), respectively. To analyze the pattern of protein hydrolysis, we used the SDS-PAGE protocol described by Laemmli [21] with 12% separating and 5% stacking gels. Samples were prepared for electrophoresis by boiling in a loading buffer containing 0.1% Coomassie Brilliant Blue R 250 dye (Sigma-Aldrich, USA). Molecular weight distribution and average molar mass were evaluated using an LC-2000 Plus HPLC system (Jasco, Japan). For evaluation, the collagen samples were prepared by filtration through a 0.45-μm membrane using a Shodex Protein KW-802.5 column (I.D. 8 mm × 300 mm; Shodex, Japan). The mobile phase used was 50 mM phosphate buffer containing 0.3 M NaCl at a flow rate of 0.9 ml/min, and the detector recorded absorbance at 220 nm. We used thyroglobulin (669 kDa), β-amylase (200 kDa), alcohol dehydrogenase (150 kDa), albumin (66 kDa), carbonic anhydrase (29 kDa), cytochrome c (12.4 kDa), aprotinin (6.5 kDa), and cyanocobalamin (1.3 kDa) as protein standards (all obtained from Sigma-Aldrich) for standard curve construction and evaluation of the average molecular weight of enzyme-hydrolyzed collagen samples.

## Results

### Elimination of Foreign Bodies in Chicken Feet

**Fig. 1:**
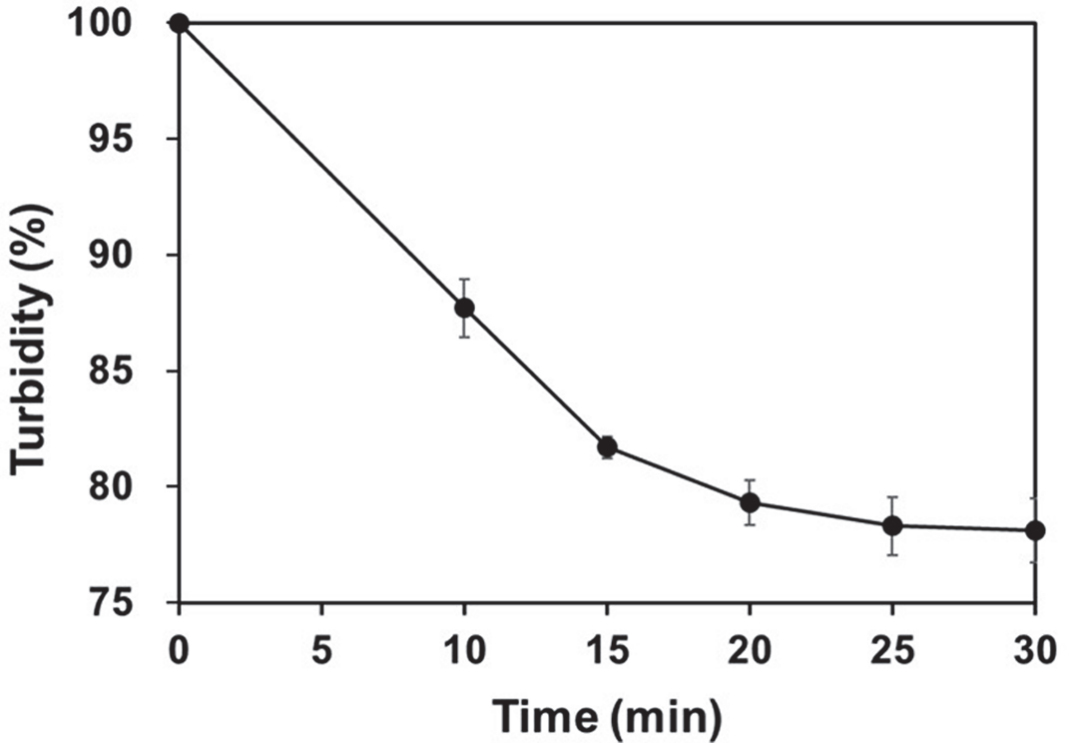
Changes in the turbidity of chicken washing water measured during different sonication periods.

The turbidity of the washing water used during ultrasonication is shown in Fig. 1. As the ultrasonication time increased from 0 to 20 min, turbidity tended to decrease rapidly and was maintained from 25 to 30 min. Widyasari and Rawdkuen [22] reported that when using ultrasonication on chicken feet, the extraction of gelatin, protein, and fat can yield results similar to acid treatment extraction, which takes a long time.

### Collagen Hot Water Extraction Time from Chicken Feet

**Fig. 2:**
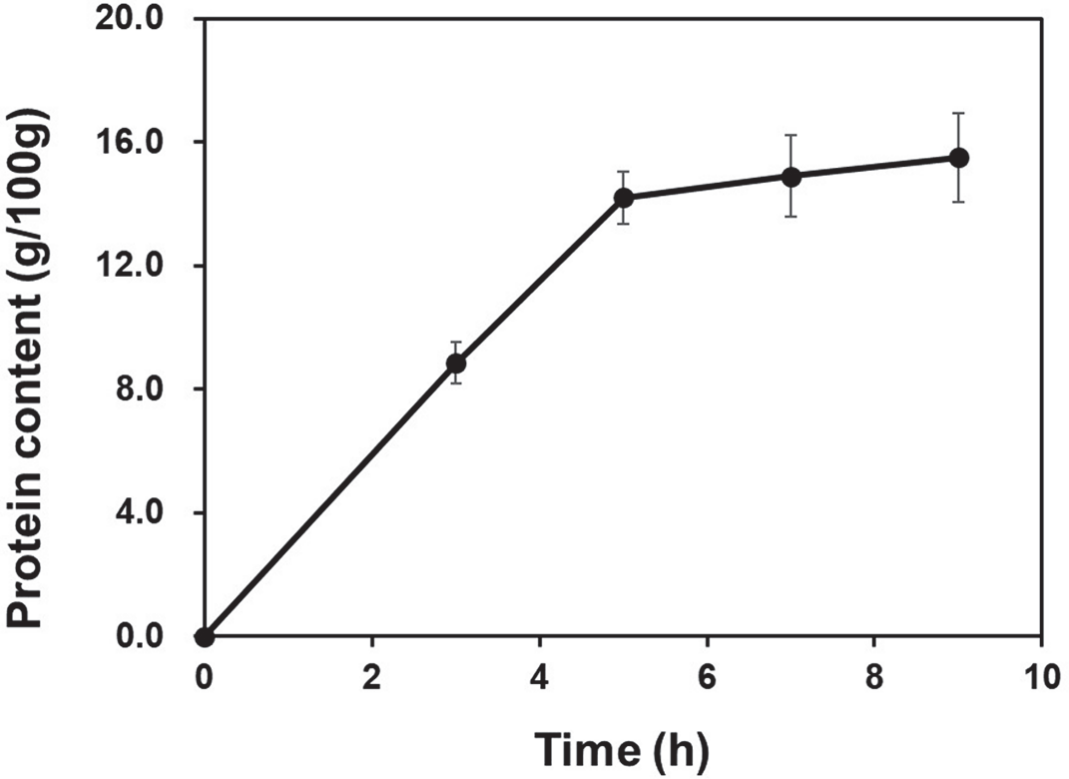
Crude protein content of chicken feet measured during different hot water extraction periods.

The hot water extraction time of collagen from chicken feet is shown in Fig. 2. The protein content of chicken feet according to the hot water extraction time increased rapidly from 0 to 5 h and then gradually increased after 5 h (14.2 g/100 g). The highest protein content was observed at 9 h of extraction (15.5 g/100 g), but there was no significant difference to that at 7 h (14.9 g/100 g) (*p* > 0.05). The crude protein and collagen contents of Korean chicken foot extract (9 h sample) were 15.5 g/100 g and 13.9 g/100 g, respectively, which compares with protein and collagen contents of 18.1% and 8.24%, respectively, as previously reported for chicken (*G. gallus domesticus*) feet by Potti and Fahad [23]. Therefore, in this study, to extract high collagen content, the 8-h treatment seems appropriate from an economical point of view.

### Hydrolytic Pattern of Enzyme-Treated Chicken Foot Collagen

**Fig. 3:**
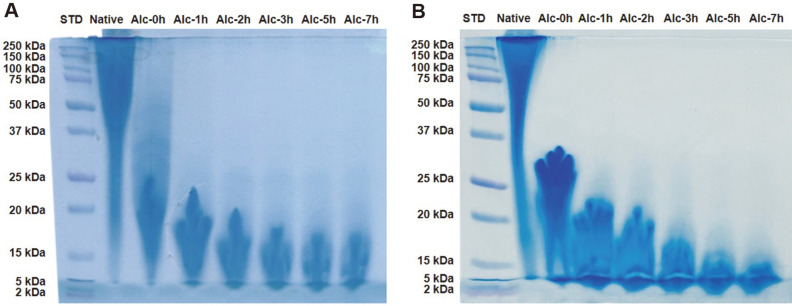
Sodium dodecyl sulfate-polyacrylamide gel electrophoresis patterns of chicken foot collagen treated with 0.1% (A) and 1% alcalase (B).

The hydrolytic pattern of enzyme-treated collagen is shown in Fig. 3. In this study, alcalase was added at concentrations of 0.1% and 1% (w/w) to chicken feet collagen extracts. For a 7-h hydrolysis period, collagen treated with 1% and 0.1% alcalase exhibited a significantly lower molecular weight range of 2-37 kDa and 2–75 kDa, respectively, than that of native collagen (25-250 kDa). Compared with the collagen extract treated with 1% enzyme, those subjected to hydrolysis with 0.1% alcalase showed a low-molecular-weight peptide content of less than 15 kDa. The lowest range of collagen peptides (2-15 kDa) was identified in samples exposed to 7-h hydrolysis at an enzyme concentration of 1% (w/w).

### Molecular Weight Distribution of Chicken Foot Collagen

**Fig. 4:**
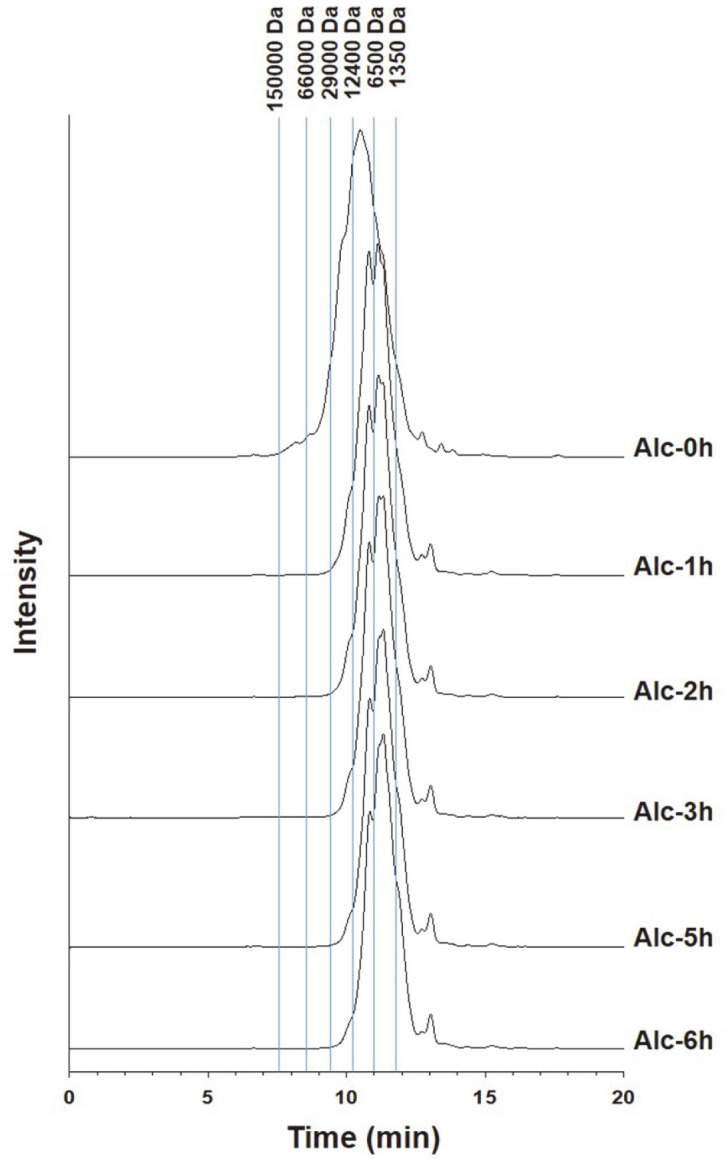
High-performance liquid chromatography analysis of the molecular weight distribution of chicken foot collagen treated with 1% alcalase.

The size-exclusion chromatographic profiles of chicken foot collagen treated with 1% alcalase are shown in Fig. 4. Results indicated a reduction in the high-molecular-weight fractions greater than 29 kDa after hydrolysis for 1 h and a concomitant increase in low-molecular-weight fractions of less than 6.5 kDa.

The effect of hydrolysis duration on the molecular weight distribution of chicken foot collagen peptides was also assessed based on the HPLC profiles, as shown in Table 1. These profiles revealed that compared with native peptides, collagen peptides significantly degraded after hydrolysis for 1 h, and the amount of peptides with molecular weights less than 5 kDa increased by 160.7%. Similarly, we observed an initial increase in the proportion of peptides in the 5–10 kDa range, with a reduction from 37.2% to 41.4% being recorded at 2 h. However, with further prolongation of hydrolysis, we detected a 34.8% reduction in peptides within this size range. In addition, the proportion of high-molecular-weight peptides (10–700 kDa) was significantly reduced to 76.8%. The average molecular weight of collagen peptides in 1-h hydrolysates was 6,088 Da, which represents a reduction of 49.8% compared with untreated collagen. Although there was a time-dependent degradation of higher molecular weight collagen peptides (10–700 kDa), a greater degradation was observed after hydrolysis for 7 h (14.9%) compared to that after 1 h. Compared with the average molecular weight of 12,131 Da recorded at 0 h, the average molecular weight of collagen hydrolyzed for 3, 5, and 7 h was 5,232, 4,929, and 4,916 Da, respectively.

## Discussion

Ultrasonication effectively removed foreign substances, such as blood, from chicken feet in a short period. The optimal condition for foreign body removal using ultrasonication was treatment at 28 kHz with 1.36 kW for more than 25 min.

Consistent with the results obtained from SDS-PAGE analysis, we observed a strong correlation between a prolonged period of enzymatic hydrolysis and low-molecular-weight peptide distribution. This association between the duration of enzymatic hydrolysis and molecular size of collagen peptides has been described previously [3]. Notably, low-molecular-weight collagen peptides generally have better bioactivities than fractions with high molecular weights [24].

Alcalase enzymes are characterized by a broad catalytic spectrum, whereas trypsin preferentially hydrolyzes peptides by targeting C-terminal lysine and arginine residues [3]. From the perspective of protein structure analysis, enzymatic hydrolysis, such as that of alcalase, contributes to the degradation of the triple-helix structure of collagen [25]. In addition, enzymatic hydrolysis generally does not require high temperatures and involves the breakage of specific peptide bonds, thereby enabling a relatively good prediction of the peptide hydrolysis profile for process standardization [24]. Given these properties, the enzymatic hydrolysis of collagen would be an efficient and controllable process that could be employed to obtain a low-molecular-weight portion of collagen, thereby potentially enhancing its bioavailability.

As the concentration of alcalase increased, low molecular weight collagen increased. These findings are consistent with those reported previously by Khiari *et al*. [3], who established that collagen peptides with lower molecular weights can be obtained using higher enzyme concentrations and longer hydrolysis times. Collagen peptide hydrolysate profiles are distinguishable by molecular weight; for example, myosin light chain (~24 kDa) and tropomyosin (~36 kDa) [3].

As the enzymatic hydrolysis time increased, the average molecular weight of collagen decreased. This profile revealed that 99.8% of total hydrolysis occurred during the initial 5 h of treatment, with only a relatively small fraction occurring in the subsequent 2 h. Needless to say, this has important commercial implications, indicating that hydrolysis conducted for 5 h would be a more economically efficient process than a 7-h treatment. These findings indicate that a proportion of high-molecular-weight collagen peptides is converted to low-molecular-weight peptides by enzymatic hydrolysis. In this context, the bioavailability of proteins and peptides is reduced when the molecular weight of these molecules exceeds 700 Da [26]. Khiari *et al*. [3] suggested that enzymatic hydrolysis of collagen may represent an effective practical means of enhancing the absorption of collagen peptides in the small intestines (*i.e.*, increasing their bioavailability).

In this study, we demonstrated that the hydrolysis of collagen using a high concentration of alcalase in conjunction with a long hydrolysis time resulted in a low-molecular-weight distribution among collagen peptides derived from Korean chicken foot extracts. Specifically, collagen peptides with the lowest average molecular weight (4,916 Da) were obtained when hydrolysis was performed for 7 h using an alcalase concentration of 1% (w/w). Considering the time- and energy-efficient processes conducive to yielding collagen peptides with low molecular weights, we propose that the application of 1% alcalase hydrolysis for up to 5 h constitutes commercially meaningful collagen hydrolysis conditions. Based on size-exclusion chromatographic profiling, we established that low-molecular-weight collagen peptides of approximately 1,000–5,000 Da were obtained after hydrolysis for 1 h in the presence of 1% alcalase. Collectively, our findings provide evidence that Korean chicken feet are a viable, alternative natural source of collagen and propose suitable conditions for its effective enzymatic hydrolysis.

## Figures and Tables

**Table 1:** Molecular weight distribution of chicken foot collagen treated with 1% alcalase.

Sample	Amount (% of integration areas^a^)				Average molecular weight (Da)

	<1 kDa	1–5 kDa	5–10 kDa	10–700 kDa	
Alc-0h	1.46	17.8	37.2	43.5	12,131
Alc-1h	2.17	46.4	41.4	10.1	6,088
Alc-2h	1.93	48.5	42.1	7.43	5,495
Alc-3h	2.05	52.3	40.1	5.61	5,232
Alc-5h	2.85	56.2	36.0	4.89	4,929
Alc-7h	3.06	58.5	34.8	3.60	4,916

^a^The amount of each molecular weight fraction was calculated as a percentage of the integration area with respect to the total peak area under the chromatogram.
